# Optical genome mapping uncovers clinically relevant structural variants in congenital heart disease with heterotaxy

**DOI:** 10.3389/fgene.2025.1673539

**Published:** 2025-12-05

**Authors:** Shaojie Min, Jingwei Sun, Weicheng Chen, Zhiyu Feng, Quannan Zhuang, Yuan Gao, Siyi Lin, Siyu Sun, Yuquan Lu, Shuolin Li, Xueying Tian, Guoying Huang, Wei Sheng, Xianghui Huang

**Affiliations:** 1 Shanghai Key Laboratory of Birth Defects, Pediatric Heart Center, Children’s Hospital of Fudan University, Shanghai, China; 2 BengBu First People’s Hospital, BengBu, China; 3 Obstetrics and Gynecology Hospital, Institute of Reproduction and Development, Fudan University, Shanghai, China; 4 Fujian Key Laboratory of Neonatal Diseases, Children’s Hospital of Fudan University at Xiamen (Xiamen Children’s Hospital), Fujian, China; 5 Research Unit of Early Intervention of Genetically Related Childhood Cardiovascular Diseases (2018RU002), Chinese Academy of Medical Sciences, Shanghai, China

**Keywords:** congenital heart disease, heterotaxy, optical genome mapping, structural variations, candidate genes

## Abstract

**Introduction:**

The genetic factors underlying congenital heart disease and heterotaxy (CHD/HTX) are complex, including copy number variants, loss-of-function mutations, and missense variants, many of which can be detected by high-throughput sequencing. The screening for chromosomal structural variations (SVs) is another important strategy to understand the genetic etiology of CHD/HTX.

**Methods:**

We employed optical genome mapping (OGM), an innovative technique capable of capturing SVs often missed by traditional cytogenetic methods, to screen for SVs in 12 patients with complex CHD/HTX. Several patients had previously undergone chromosomal microarray analysis (CMA) or whole exome sequencing (WES), but their genetic diagnoses remained inconclusive.

**Results:**

By integrating data from CMA or WES, we analyzed potentially pathogenic SVs in patients with CHD/HTX. In total, we identified 825 high-confidence SVs, including 609 SVs (73.7%) located in intergenic regions or containing introns, pseudogenes, or RNA genes, while 217 (26.3%) overlapped the coding regions of genes. Analyzed through AnnotSV, DECIPHER and OMIM databases, 7 SVs of interest were identified, including: one previously reported pathogenic SV, three SVs overlapping established CHD/HTX associated genes (*NOTCH2*, *KDM6A* and *CBL*), and three SVs located in *SMARCA2* and *CEP164*, which are proposed as candidate susceptibility genes pending further validation.

**Conclusion:**

Our findings highlight the utility of OGM in analyzing the genetic etiology of CHD/HTX and contribute to broadening of the complex genetic landscape underlying these diseases.

## 1 Introduction

Congenital heart disease (CHD) is the most common birth defect affecting approximately 8.98‰ of live births in China (Zhao et al., 2019). Heterotaxy (HTX) is a rare disorder characterized by the abnormal arrangement of visceral organs along the left-right (L-R) body axis and is strongly associated with complex CHD. Approximately 83% of HTX patients present with complex CHD, including double outlet right ventricle (DORV), transposition of the great arteries (TGA) and atrioventricular septal defects. Conversely, about 2.3% of patients with CHD are diagnosed with HTX, a condition associated with poor prognosis and high mortality (Lin et al., 2014; Huang et al., 2023).

The etiology and underlying mechanisms of the co-occurrence of CHD and HTX remain incompletely understood despite decades of research. Bilateral symmetry in the embryo is first broken at the ventral node (also referred to as the left–right organizer, LRO), where motile cilia generate a leftward directional flow (nodal flow) through their posteriorly tilted rotational motion. In turn, immotile cilia sense this flow and transmit asymmetric signals to the lateral plate mesoderm (LPM), leading to asymmetric gene expression (Hirokawa et al., 2006; Yoshiba et al., 2012). To date, causative genetic mutations identified in CHD/HTX patients often involve genes related to L-R asymmetric development (e.g., *NODAL* (Dardas et al., 2024), *ZIC3* (Ware et al., 2004)) and ciliary assembly and function (e.g., *DNAH11* (Liu et al., 2019), *DNAH5* (Bolkier et al., 2022), *DNAI1* (Kennedy et al., 2007)). Additional clinically significant genes have also been reported, including *PKD1L1* (Vetrini et al., 2016), *SHROOM3* (Tariq et al., 2011), *MMP21* (Guimier et al., 2015), *PNPLA4* (Gao et al., 2024). However, these mutations explain only a small fraction of CHD/HTX cases, leaving the genetic etiology of the majority of patients unresolved.

Structural variations (SVs) are genomic rearrangements of at least 50 bp in length, including deletions, duplications, insertions, translocations and copy number variations (CNVs) (Sudmant et al., 2015). SVs have long been recognized as major contributors to human diseases, particularly genetic disorders (Weischenfeldt et al., 2013). Numerous studies have demonstrated a substantial burden of CNVs in CHD/HTX. For example, Glessner JT et al. analyzed a cohort of 538 CHD trios and found that the frequency of *de novo* CNVs among patients with heterotaxy was as high as 21% (Glessner et al., 2014). Similarly, another study identified clinically relevant CNVs in approximately 20% of patients with CHD/HTX (Cowan et al., 2016). However, these studies primarily employed chromosomal microarray analysis (CMA), which has limitations in the detection of balanced SVs and resolution. Therefore, the true burden of SVs in CHD/HTX patients remains significantly underexplored.

Optical genome mapping (OGM) is a novel, high-resolution (>500 bp) and genome-wide technique that does not rely on DNA amplification. It enables the detection of all classes of SVs, including CNVs, balanced translocations and inversions in a single test. An increasing number of studies have confirmed that OGM shows high concordance with traditional detection techniques for detecting SVs, while also revealing additional clinically relevant SVs, thereby advancing the field of precision medicine. However, the clinical application of OGM in birth defects remains limited. In this study, genomic SVs in CHD/HTX samples were analyzed by OGM.

## 2 Materials and methods

### 2.1 Patient sample collection

A total of 12 peripheral blood samples were collected from patients with CHD/HTX and stored at −80 °C in accordance with the Helsinki Declaration. All participants were of Chinese origin. Optical genome mapping analysis was performed on all de-identified samples by two independent operators. Some cases had previously undergone WES (n = 2) and CMA (n = 1). This study was approved by the Ethics Committee of Children’s Hospital of Fudan University.

### 2.2 De novo assembly and structural variant calling and filtering

OGM was performed as previously described (Neveling et al., 2021). Briefly, ultra-high-molecular-weight (HMW) DNA was extracted from frozen peripheral blood using Bionano SP Blood & Cell Culture DNA Isolation Kit, and then HMW DNA was labelled at the specific sequence CTTAAG with the Direct Label and Stain (DLS) Kit according to the manufacturer’s protocol. The labelled HMW-DNA was loaded onto Bionano Genomics Saphyr Chip for electrophoretic linearization and imaging.

*De novo* assembly pipeline was executed using Bionano Solve (version 3.7) with the human reference genome GRCh38 as the reference. For SV calls filtering, the following criteria were applied: (1) recommended confidence with values: insertions and deletions >0, inversions >0.7, duplications = 1, intrachromosomal fusions >0.05, CNV > 0.99; (2) recommended sizes: insertions and deletions >500 bp, CNV > 500 Kbp, AOH/LOH >25,000 Kbp; (3) SVs occurring in ≤1% of Bionano control samples (n > 300). All data were visualized using Bionano Access (version 1.7 or version 1.7.2). To identify clinically relevant SVs, only those overlapping the coding regions of genes were selected. The remaining SVs were analyzed by AnnotSV and interpreted by OMIM (https://omim.org/), gnomAD (https://gnomad.broadinstitute.org/), DGV (http://dgv.tcag.ca/dgv/app/home) and DECIPHER (https://www.deciphergenomics.org/) databases.

### 2.3 Whole exome sequencing and chromosomal microarray analysis

Genomic DNA was extracted from peripheral blood samples using the QIAamp DNA Blood Mini Kit (Qiagen) following the manufacturer’s instructions. Whole exome sequencing (WES) was performed on an Illumina HiSeq X Ten platform. Detailed methodologies for exome library preparation, sequencing and variant calling were performed as previously described (Wang et al., 2025). We screened for known pathogenic variants or rare deleterious coding variants (MAF < 0.0001 in gnomAD_exome_EAS and CADD score >20). Additionally, patient 2247 underwent chromosomal microarray analysis (CMA) following previously published protocols (Gao et al., 2024).

### 2.4 Literature review

A literature review was conducted through PubMed search using the keywords “Optical Genome Mapping OR OGM” and “Structural variation OR Structural variant OR SV”. Only studies focusing on the clinical application of OGM in human samples and comparing OGM with standard cytogenetic techniques were systematically reviewed. Detailed information is summarized in Table 1.

**TABLE 1:** Clinical features of patients with complex CHD and heterotaxy.

Case	Sex	Age	Cardiovascular malformations	Other malformations
1809	M	7y2m	L/TGA	/
2247^a^	F	2y10m	Dextrocardia, single ventricle, pulmonary atresia	/
2582^b^	M	1y2m	Atrial situs ambiguity, single atrium, single ventricle, pulmonary atresia	/
2765	F	4y2m	Dextrocardia, single atrium, single ventricle, DORV, ASD, pulmonary stenosis	/
2962	F	0d	SI, VSD	/
3083	F	7m2d	Isolated levocardia, DORV, VSD, aorta anterior/IVC right of spine	/
3120	F	9y3m	Dextrocardia, functional single ventricle (tricuspid atresia), TGA, ASD, pulmonary stenosis	Shortened and partially absent right fourth rib
3167	M	6d	Dextrocardia, TGA, PDA, ASD, SI	Polydactyly, right accessory auricle, spine malformation
3718	M	4d	Isolated levocardia, TGA, VSD, ASD, pulmonary stenosis, right superior vena cava draining into the left atrium	/
3861	M	6y3m	TOF, left atrial isomerism	Pulmonary isomerism
3924	M	5y2m	Right atrial isomerism, single ventricle, AVSD, DORV, TGA, TAPVC, pulmonary stenosis, anomalous pulmonary venous drainage, right-side aortic arch	Asplenia
4066	F	3y2m	Isolated levocardia, DORV, VSD, ASD, pulmonary stenosis	/

ASD, atrial septa defect; AVSD, atrioventricular septal defect; DORV, double outlet right ventricle; IVC, inferior vena cava; L/TGA, L-transposition of the great arteries; PDA, patent ductus arteriosus; SI, situs inversus; TAPVC, total anomalous pulmonary venous connection; VSD, ventricular septal defect.

^a^ This patient underwent both CMA, and WES, but no pathogenic variants or likely pathogenic variants were identified.

^b^ This patient underwent WES, but no pathogenetic or likely pathogenic variants were identified.

## 3 Results

### 3.1 Patient characteristics

Our study enrolled 12 patients with complex CHD and heterotaxy, including 6 females and 6 males. Levocardia was observed in 58% (7/12), dextrocardia in 42% (5/12). The mean age at diagnosis was 3.3 years (range 0–9.3 years), and all complex cardiac defects were diagnosed by echocardiogram. Patient 2962 died of respiratory failure at the age of 4 months and 8 days. In addition to cardiovascular malformations, four patients presented with other structural anomalies, such as asplenia and polydactyly. Case 2247 underwent CMA, which did not identify any pathogenic, likely pathogenic CNVs or variants of uncertain significance (VUS). WES was also performed for this patient, but the result was inconclusive; therefore, no further analysis was conducted. Case 2582 also underwent WES, which did not reveal any clinically relevant variations. Detailed clinical characteristics of all patients are summarized in Table 1.

### 3.2 Raw data quality and SVs detected by OGM

Bionano optical genome imaging generated an average of 570.9 Gb of raw data per sample. The 12 samples achieved an average effective coverage of 124.74X (range: 92.58-232.21X) and a map rate of 89.31% (±3.05%). The label density ranged from 14.45–16.56 per 100 kb, and the range of N50 molecular length (≥150 kb) was 260.63–367.39 kb. Thus, all samples passed the quality control (QC) metrics. Detailed QC parameters for each sample are listed in Supplementary Table S1.

**FIGURE 1:**
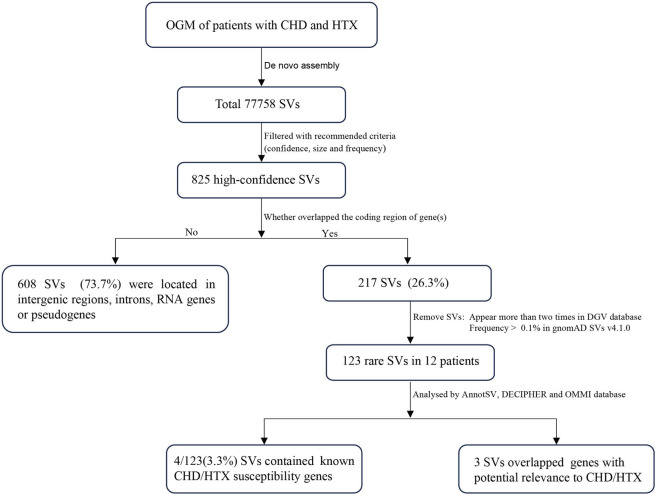
The workflow of this study.

**FIGURE 2:**
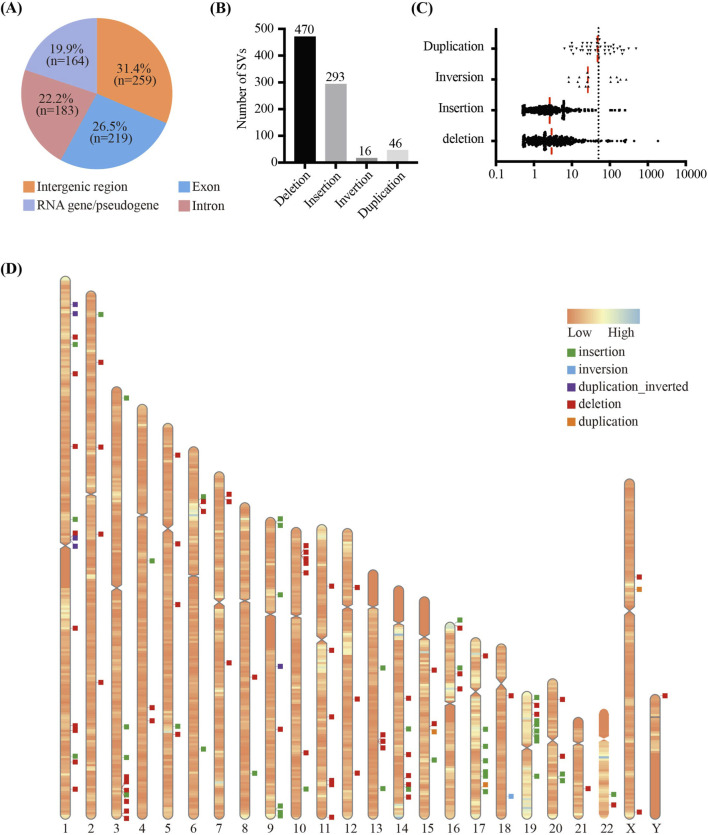
Distribution of high confidence SVs in 12 patients with complex CHD and HTX **(A)** Summary of proportion of SVs located in intergenic regions, RNA genes or pseudogenes, exons and introns. **(B)** The distribution of detected SVs (deletions, insertions, inversions, duplications). **(C)** The scatter plot illustrates the length distribution of the identified SVs. Each dot represents one SV. The dashed line marks the 50 kb threshold. The red vertical lines represent the median values. **(D)** The distribution of 123 rare SVs on chromosomes. Colored boxes on the right side of the chromosome represent the SV types, including deletion, insertion, duplication, inversion and duplication_inverted respectively.

In total, 77758 SVs were detected in 12 samples (Figure 1). After filtering out low-confidence and common SVs, 825 SVs (1.1%) remained, with an average of 68.8 SVs per sample (including 39.2 deletions, 24.4 insertions, 1.3 inversions and 3.8 duplications). SVs were detected in all chromosomes, with the highest SV burden observed on chromosomes 1 (Supplementary Figure S1). The majority of SVs did not overlap any coding gene, and only 26.3% of SVs overlapped one or more exons of protein coding gene(s), potentially affecting coding sequences (Figures 1, 2). Among all SV types, deletions were the most frequent (median = 39, range: 3–44), followed by insertions (median = 24, range: 2–20) (Figure 2B). Most deletions and insertions were less than 50 kb (Figure 2C), which is consistent with recent findings from OGM-based SV detection in cancer samples (Wagener et al., 2024).

### 3.3 Detection of SVs encompassing clinically relevant genes in CHD/HTX patients by OGM

Among all high confidence SVs, we identified 216 SVs involving coding regions of genes in 12 patients. To further filter out common SVs, we removed SVs that appear more than twice in DGV (overlapping more than 50%) and frequency >0.1% in gnomAD, leaving only 123 rare SVs for further clinical significance assessment. The chromosomal distribution of these rare SVs is shown in Figure 2D. Among them, seven SVs of interest were identified (Supplementary Table S2): one with previously established clinical significance, three involving known CHD/HTX susceptibility genes, and three affecting novel candidate genes.

#### 3.3.1 Known pathogenic SVs and SVs harboring known CHD/HTX susceptibility genes

**FIGURE 3:**
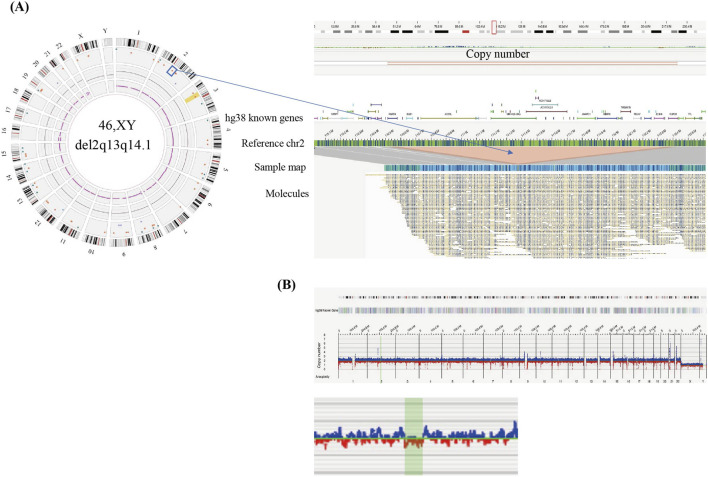
Identification of a chromosome 2q deletion in sample 1809 by OGM **(A)** Left panel shows a circos plot illustrating SVs in the sample and the blue box on chromosome 2 highlights the pathogenic deletion. Right panel representing the genome browser view provides the detail of the structural variation. The sample’s map (blue bar) alignment to the reference map of chromosomes 2 reveals a large ∼1.85 Mbp deletion (light red) and mapping genes in GRCh38/hg38. **(B)** Copy number profile of chromosomes 2 indicates a loss in chromosome 2q.

A 1.85 Mb heterozygous deletion in 2q13 region was identified in patient 1809 (Figure 3). The deletion encompasses 11 protein-coding genes, including *RGPD6*, *BUB1*, *ACOXL*, *BCL2L11*, *ANAPC1*, *MERTK*, *TMEM87B*, *FBLN7*, *ZC3H8*, *ZC3H6* and *RGPD8*. The cardiovascular phenotype associated with 2q13 deletion syndrome has been reported as highly variable (Digilio et al., 2022). In our study, the proband 1809 presented with L-transposition of the great arteries (L-TGA), a condition not previously reported in 2q13 deletion cases. According to ACMG guidelines, this variant was classified as pathogenic.

**FIGURE 4:**
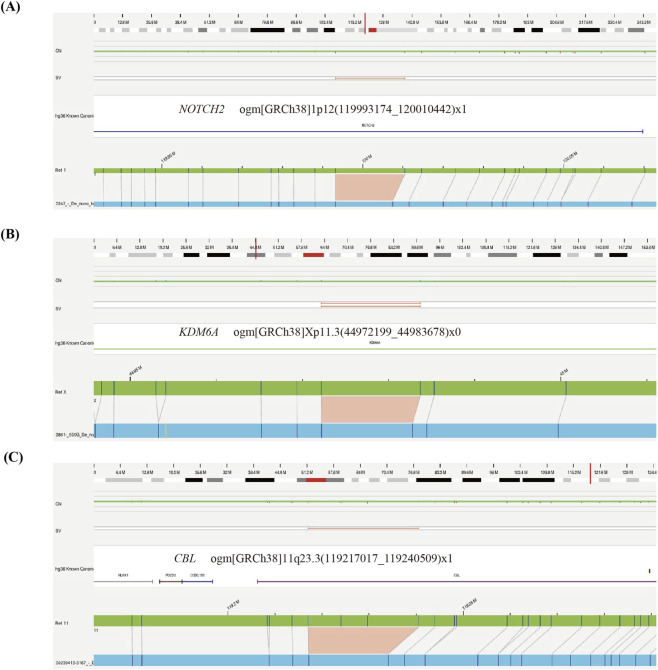
Genome browser view of three SVs overlapping CHD/HTX susceptibility genes: **(A)** NOTCH2. **(B)** KDM6A. **(C)** CBL. The green bar represents the reference map, while patient maps are shown in blue. Deletions are highlighted in light red, and insertions are marked in light blue.

In addition to the above pathogenic SVs, we identified three SVs encompassing three known syndromic CHD-associated genes (Figures 4A–C): *NOTCH2* (#MIM 610205), *KDM6A* (#MIM 300867), and *CBL* (#MIM 613563). These included a 3.3 kb deletion of 1p12 region overlapping exon3 and exon4 of the *NOTCH2* gene in patient 2247, who had previously undergone CMA but no clinical CNVs were found; a deletion of Xp11.3 region potentially disrupting exon4 of *KDM6A* gene in case 3861; and a 6.6 kb heterozygous deletion of 11q23.3 in case 3617, partially deleting the *CBL* gene. Both patients 3861 and 3617 presented with syndromic CHD.

#### 3.3.2 OGM unveiled rare SVs involving candidate susceptibility genes

**FIGURE 5:**
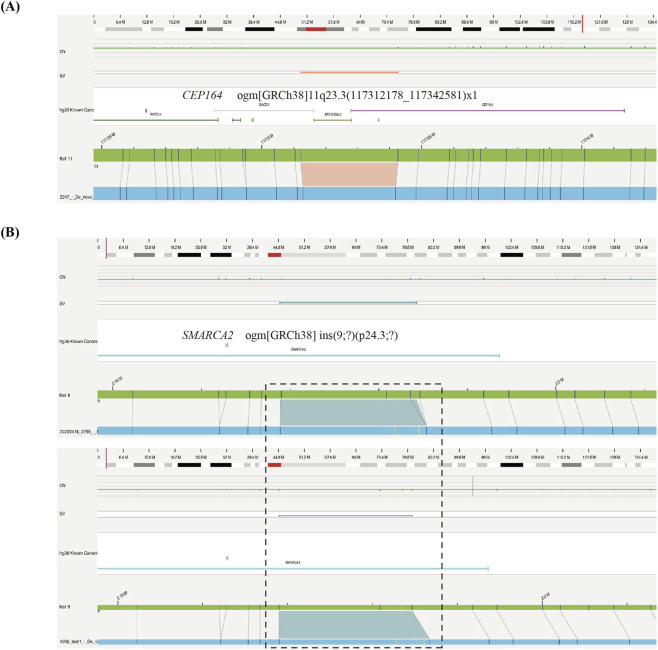
Genome browser view of SVs overlapping potential CHD/HTX candidate genes (*CEP164* and *SMARCA2*) **(A)** A 1.4 kb deletion of *CEP164* on 11q13.3. **(B)** Heterozygous insertions in the *SMARCA2* gene in two patients (case 2765 and 4066).

Through a comprehensive analysis of the remaining rare SVs, we identified three SVs of interest that potentially overlap novel candidate genes of CHD/HTX in three patients, including *CEP164* and *SMARCA2*. Patient 2247 carried a unique 1.4 kb deletion, ogm [GRCh38] 11q23.3 (117312178_117342581)x1 (Figure 5A). This deletion partially affects the 5′UTR and exon 1-3 of *CEP164* gene and is predicted to result in loss of function. In two unrelated patients 2765 and 4066, we found 1035 bp and 2049 bp insertions respectively in 9p24.3 (Figure 5B), which may disrupt *SMARCA2* function. Both patients presented with complex CHD, including DORV, pulmonary stenosis, ASD, single atrium and single ventricle.

The expression patterns of the two candidate genes in the developing human heart were analyzed by publicly available single cell RNA-seq data (Speir et al., 2021). As shown in Supplementary Figure S2A–C, both *CEP164* and *SMARCA2* are widely expressed in the human embryonic heart, with *SMARCA2* exhibiting particularly high expression levels. Although their expression is relatively low in cardiomyocytes, they exhibit higher expression levels in non-cardiomyocyte cells, such as fibroblasts and smooth muscle cells. Consistent with these observations, *in situ* hybridization data obtained from the GenePaint database (https://www.genepaint.org) revealed similar expression patterns in E14.5 mouse embryos, with both *Cep164* and *Smarca2* being expressed in the developing heart and surrounding tissues (Supplementary Figure S2D,E). In addition, a previous study employing wholemount β-galactosidase (LacZ) staining demonstrated broad *Cep164* expression during early murine embryogenesis (E9.5, E10.5, and E12.5), including the central ventricle, bulbus cordis, and outflow tract of the developing heart (Devlin et al., 2020). Taken together, these findings provide converging evidence that *CEP164* and *SMARCA2* may represent plausible contributors to CHD/HTX pathogenesis and warrant further functional investigation.

## 4 Discussion

SVs play a critical role in a variety of diseases, including CHD/HTX. Current methods for detecting SVs mainly include karyotype (KT), FISH and CMA. However, each technique has its own limitations. KT requires cell culture and has a low resolution of approximately 5–10 Mb. FISH is a targeted approach limited to the detection of known variations. CMA is unable to detect balanced chromosomal aberrations (e.g., inversions, translocations) and determine the precise location/orientation of insertions. Given these limitations, the true burden of SVs may be significantly underestimated in CHD/HTX, highlighting the need for higher-resolution genome-wide approaches to elucidate its genetic architecture.

OGM represents a genome-wide, high-resolution platform for SV detection that bridges the gap between cytogenetic and sequencing approaches. Recent studies have evaluated the performance of OGM across diverse clinical settings, including hematologic malignancies, solid tumors, prenatal diagnosis, reproductive disorders, and neurodevelopmental diseases (Xiao et al., 2024; Schrauwen et al., 2024; Cheng et al., 2024; Shim et al., 2022; Dai et al., 2022; Loghavi et al., 2024). These studies have consistently demonstrated high concordance (frequently approaching 100%) with conventional cytogenetic methods, while revealing additional clinically relevant SVs that were undetectable by CMA or short-read sequencing (Supplementary Table S3). Although these findings firmly establish OGM as a next-generation cytogenomic tool, its utility in congenital heart disease, particularly in CHD/HTX, remains largely unexplored.

Building upon previous validations of OGM, we applied this technology to investigate the cryptic SV burden in CHD/HTX. In this study, most SVs identified by OGM were less than 50 kb in size, which were frequently elusive to conventional cytogenetic methods. The smaller size of these SVs facilitates the prioritization of potential candidate genes. Additionally, most SVs were located in non-coding regions, requiring integrative analyses with complementary approaches to assess their clinical relevance. OGM revealed a total of 7 SVs potentially associated with the patients’ phenotypes, including one microdeletion of known pathogenicity, three overlapping known CHD susceptibility genes, and three overlapping candidate susceptibility genes. Among them, the SVs involving *CEP164* and *SMARCA2* are currently lacking established clinical significance, but these findings may provide valuable information for future research.

The recurrent 2q13 deletion syndrome is associated with developmental delay and multiple deformities, including cranial dysmorphism, CHD and urogenital malformations (Yu et al., 2012; Riley et al., 2015; Wolfe et al., 2018). CHD has been reported in approximately 30%–60% of affected individuals, including diverse manifestations such as heterotaxy, tetralogy of Fallot (TOF), and septal defects (Digilio et al., 2022). Zebrafish models have implicated *FBLN7* and *TMEM87B* as candidate genes for cardiac defects and craniofacial abnormalities (Russell et al., 2014). However, the specific molecular mechanisms underlying their roles in cardiogenesis remain unclear. Notably, a potentially deleterious *TMEM87B* variant was identified in a patient with a hemizygous 2q13 microdeletion, suggesting a recessive condition characterized by CHD and restrictive cardiomyopathy (Yu et al., 2016). In our cohort, a patient carrying a 2q13 microdeletion was diagnosed with isolated CHD-L/TGA. This finding is consistent with a previous study describing a patient with 2q13 deletion syndrome who also exhibited CHD and heterotaxy, further supporting a role for this region in left-right patterning (Rudd et al., 2009).

Several studies have demonstrated that OGM can achieve molecular diagnosis in cases unresolved by conventional cytogenetic techniques and short-read sequencing. In this study, we applied OGM to investigate SVs in two unexplained CHD/HTX cases. Case 2247 had previously undergone CMA, which did not reveal any clinically relevant SVs. However, OGM identified two SVs in this patient: a likely pathogenic 3.3 kb deletion in the 1p12 region overlapping the CHD-associated gene *NOTCH2*, and a 1.4 kb heterozygous deletion of uncertain significance affecting part of the 5′UTR and exons 1-3 of *CEP164*, which encodes a protein required for primary cilia assembly. Consistent with previous functional studies, zebrafish experiments have shown that *CEP164* deficiency results in ciliopathy phenotypes, including abnormal heart looping (Chaki et al., 2012). Additional parental genotyping and RNA-seq analyses are necessary to clarify the pathogenic role of these SVs. In another WES-negative patient (ID 2582), 16 rare SVs were identified; however, none were classified as pathogenic or likely pathogenic, suggesting that SVs may not represent the primary causative factor in this case.

In addition, two insertions within the *SMARCA2* gene at 9p24.3 were identified in two unrelated patients and were absent in the OGM control database. *SMARCA2* encodes a subunit of the ATP-dependent chromatin remodeling complex SWI/SNF, which regulates chromatin structure and transcription and plays a crucial role in cardiac development. Mutations in *SMARCA2* were reported in developmental syndromes that involved CHD, including Nicolaides–Baraitser syndrome (#MIM 601358) and blepharophimosis intellectual disability syndrome (Cappuccio et al., 2020). Moreover, the 9p24.2 locus (rs7863990, close to *SMARCA2*) has been identified as CHD risk loci in Chinese populations (Lin et al., 2015). Homozygous ablation of *Smarca2* in mouse embryonic stem cells resulted in impaired cardiac differentiation, and instead, cells acquired a neuronal identity (Hota et al., 2022). To our knowledge, this is the first report of SVs involving *SMARCA2* in CHD/HTX patients, providing new insights into its role in cardiac development.

## 5 Limitation

Although OGM is a comprehensive technique for detecting SVs, it has some limitations. OGM relies on fluorescent labels at specific sequences rather than DNA sequencing, making it unable to detect single nucleotide variants or poorly labeled regions, such as centric fusions, centromeres and telomeres. Moreover, a reliable control database for interpreting SVs, especially those <50 kb in size, is currently lacking, which poses a challenge for interpreting novel or rare SVs.

This study also has some limitations. First, the sample size was relatively small, and further larger cohorts are needed to clarify the correlation between candidate SVs and CHD/HTX. Second, due to insufficient blood samples from probands’ parents, we were unable to determine the inheritance pattern of the identified SVs (inherited or *de novo*). Moreover, because only a few patients had undergone CMA or WES prior to OGM analysis, systematic comparison across methods was not feasible. Once additional samples become available, future studies will aim to validate the identified SVs and clarify their pathogenic relevance. Third, we did not validate the SVs by another assay such as CMA or long-read sequencing techniques. Nevertheless, the clinical utility of OGM has been well established, and it may serve as a first-tier test, replacing traditional cytogenetic tools in both prenatal and postnatal settings (Valkama et al., 2023; Sahajpal et al., 2023; Iqbal et al., 2023). Although long-read sequencing was not employed in our study, a previous study combining OGM and long-read sequencing technologies for pathogenic SVs detection in Parkinson’s disease-related iPSC has shown that triplication, duplication and large deletion were missed by long-read sequencing technologies (Trinh et al., 2024).

## 6 Conclusion

To our knowledge, this is the first study to evaluate the burden of SVs using OGM in patients with CHD/HTX. OGM detected additional SVs not captured by CMA or WES, thereby expanding the spectrum of detectable genomic alterations and providing preliminary evidence for candidate loci that may contribute to CHD/HTX. These results enhance our understanding of the genetic architecture of CHD/HTX and highlight OGM as a powerful and promising tool in rare disease genomics.

## Data Availability

The datasets presented in this article are not publicly available due to privacy and ethical restrictions involving human participants. Processed data supporting the conclusions of this study are included in the article and Supplementary Material. Requests for access to additional data can be directed to the corresponding author and will be considered in accordance with institutional ethical guidelines.
