# Supplementary material for: Optical genome mapping uncovers clinically relevant structural variants in congenital heart disease with heterotaxy
Source: Front Genet. 2025 Dec 5;16:1673539. doi: 10.3389/fgene.2025.1673539 (PMC12715421; doi:10.3389/fgene.2025.1673539)
Supplement: Supplementary file 1 [file DataSheet1.docx]

Supplementary Material

# Supplementary Figures and Tables

## Supplementary Figures

##

**Supplementary Figure 1.** The distribution of high-confidence SVs on chromosomes. Each bar represents the total number of SVs detected per chromosome, categorized by variant type: duplications (orange), inversions (blue), insertions (green), and deletions (red).


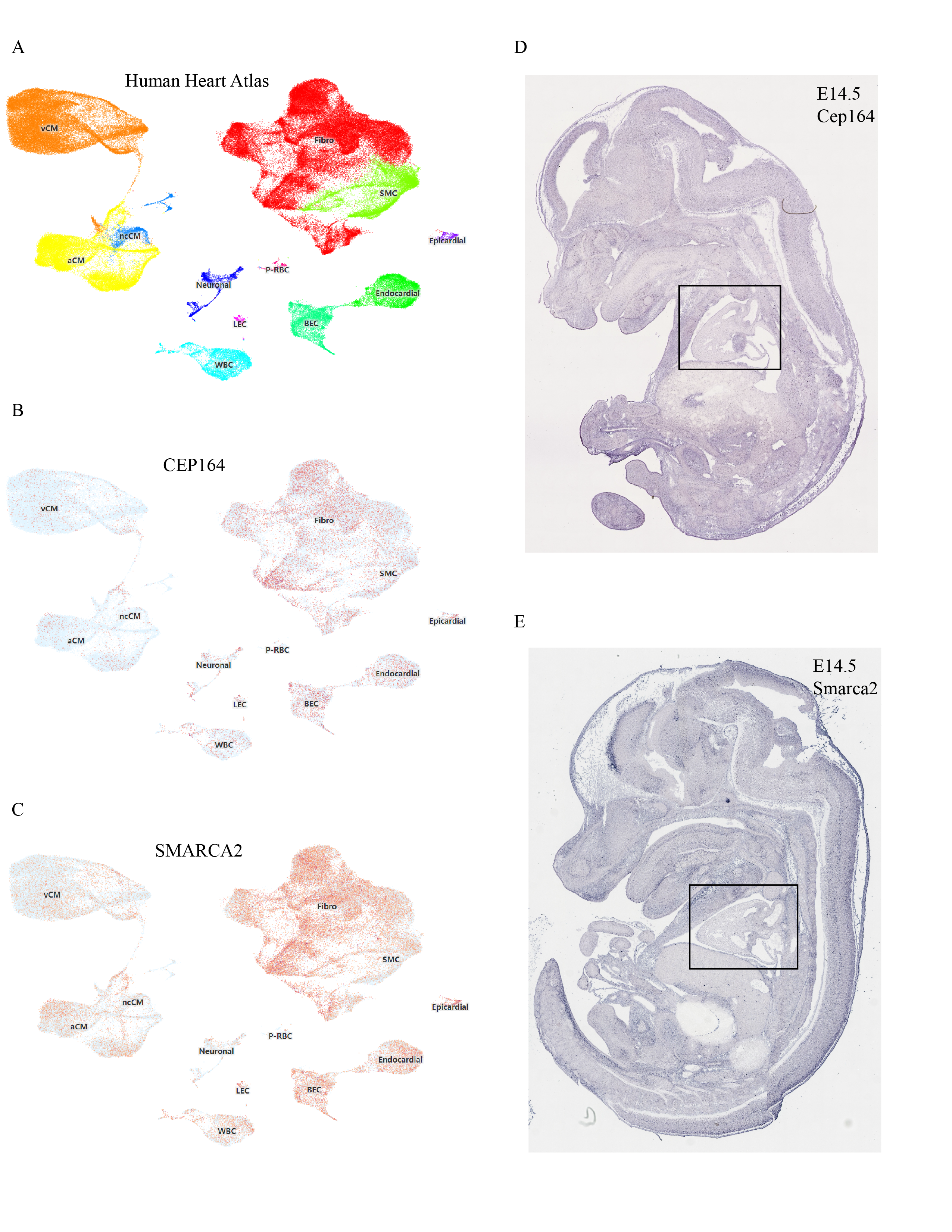


**Supplementary Figure 2.** Expression of *CEP164* and *SMARCA2* in human and mouse heart. (A-C) Single cell RNA-sequencing dataset of human heart between 9 and 16 post conception weeks obtained from the UCSC cell browser (https://cells.ucsc.edu/?ds=hoc+all-heart). (**A**) Uniform manifold approximation and projection (UMAP) visualization showing the cellular composition of the human heart. (**B**) (**C**) respectively show the expression of *CEP164* and *SMARCA2* genes in human heart atlas. (D-E) Representative in situ hybridization images of Cep164 (D) and Smarca2 (E) in E14.5 mouse embryos, obtained from the GenePaint database (<https://www.genepaint.org>). The boxed area indicated cardiac regions with gene expression signals.

## Supplementary Tables

**Supplementary Table 1 Details of OGM results**

| Patient | Total DNA(≥20 kbp, Gbp) | N50 (≥ 20 kbp, kbp) | Total DNA ((≥150kbp, Gbp) | N50 (≥ 150 kbp, kbp) | Average label density (≥150 kbp, /100Kbp) | Effective coverage X | Map rate % (≥150 kbp) |
| --- | --- | --- | --- | --- | --- | --- | --- |
| 1809 | 499.78 | 260.85 | 423.37 | 289.5 | 14.45 | 114.11 | 87.2 |
| 2247 | 399.57 | 287.25 | 337.74 | 329.25 | 15.29 | 95.91 | 91.7 |
| 2582 | 499.67 | 306.52 | 447 | 331.88 | 14.73 | 123.69 | 89.7 |
| 2765 | 1,140.61 | 241.5 | 814.89 | 315.75 | 15.13 | 232.21 | 92.1 |
| 2962 | 943.17 | 240.75 | 672.01 | 316.5 | 16.51 | 184.45 | 88.3 |
| 3083 | 667.58 | 195.13 | 417.09 | 282.75 | 16.25 | 115.72 | 89.2 |
| 3120 | 500.09 | 271.5 | 405.07 | 307.37 | 14.84 | 112.8 | 86 |
| 3167 | 499.96 | 257.63 | 419.33 | 290.25 | 15.95 | 116.76 | 89.7 |
| 3718 | 400.1 | 346.13 | 368.17 | 367.39 | 14.97 | 103.46 | 91.1 |
| 3861 | 500.04 | 240.6 | 392.77 | 290.67 | 16.56 | 105.71 | 86 |
| 3924 | 400.09 | 236.25 | 333.12 | 260.63 | 14.9 | 92.58 | 89.8 |
| 4066 | 399.55 | 263.63 | 350.74 | 284.5 | 15.38 | 99.44 | 90.9 |
| Average | 570.85 | 262.31 | 448.44 | 305.54 | 15.41 | 124.74 | 89.31 |

**Supplementary Table 2 Detailed information on seven SVs of interest**

| Patient No | Type | Location | Size(bp) | Candidate genes | Zygosity | ACMG (AnnotSV) |
| --- | --- | --- | --- | --- | --- | --- |
| 1809 | deletion | chr2:110,514,327 - 112,427,371 | 1,854,674 | *TMEM87B, FBLN7* | heterozygous | 5 |
| 2247 | deletion | chr1:119,993,174 - 120,010,442 | 3,027 | *NOTCH2* | heterozygous | 4 |
| 3167 | deletion | chr11:119,217,017 - 119,240,509 | 6615 | *CBL* | heterozygous | 4 |
| 3861 | deletion | chrX:44,972,199 - 44,983,678 | 905 | *KDM6A* | homozygous | 4 |
| 2247 | deletion | chr11:117,312,178 - 117,342,581 | 1432 | *CEP164* | heterozygous | 4 |
| 2765 | insertion | chr9:2,168,844 - 2,184,292 | 1,035 | *SMARCA2* | heterozygous | NA |
| 4066 | insertion | chr9:2,169,016 - 2,184,663 | 2,049 | *SMARCA2* | heterozygous | NA |

**Supplementary Table 3** **Systematic overview of OGM applications and comparison of conventional cytogenetic methods with OGM**

| No. | Study population/ Sample size (n) | Study design^a^ | Sample type | Conventional method^b^ | Concordance (%) | Additional findings | Ref. |
| --- | --- | --- | --- | --- | --- | --- | --- |
| 1 | Hematologic malignancies/ 52 | Retro | Bone marrow or peripheral blood | KT, FISH, CMA | 100 | ND | (1) |
| 2 | B or T-ALL)/ 10 | Retro | Bone marrow | KT, FISH, CMA, MLPA | 90 | 12 additional anomalies such as LMO2-TRA and MYC-TRB fusions | (2) |
| 3 | ALL/ 41 | Retro (38); Prosp (3) | Bone marrow or peripheral blood | KT, FISH, MLPA, RT-PCR | 100 | 10 recurrent SVs | (3) |
| 4 | AML or MDS/ 27 | Prosp | Bone marrow or peripheral blood | KT, FISH, PCR panels, CNV | 93 | 61 additional variants and refine the karyotype of 67% of samples | (4) |
| 5 | AML or MDS/ 68 | Retro (26); Prosp (42) | Bone marrow or peripheral blood | KT, FISH | 85 | Provided additional aberrations in 33%/54% of MDS/AML and successful analysis in the 3 cases of KT | (5) |
| 6 | Hematologic malignancies/ 59 | Retro | Bone marrow | KT, FISH | 99.2 | 69 translocations and 38 potential gene fusions in 45 simple cases; 8 chromoanagenesis events (4 cases), 148 translocations, and 68 potential gene fusions in 14 complex cases | (6) |
| 7 | AML/ 100 | Retro | Peripheral blood or bone marrow | KT, FISH, CMA | 100 | Provided clinically relevant information missed by the routine methods in 13% of cases | (7) |
| 8 | BCP-ALL/ 60 | Prosp | Bone marrow or peripheral blood | KT, FISH, SNP array, WES | 95 | 677 SVs, of which 66% (448/677) were focal deletions with a median of 40.5kb | (8) |

**Supplementary Table 3 continued**

| No. | Study population/ Sample size (n) | Study design^a^ | Sample type | Conventional method^b^ | Concordance (%) | Additional findings | Ref. |
| --- | --- | --- | --- | --- | --- | --- | --- |
| 9 | Hematologic malignancies/ 27 | Retro | Bone marrow | FISH, CMA, RNA fusion panel and RT-PCR | 76 | Revealed additional SVs, including submicroscopic SVs and novel fusions, in five cases | (9) |
| 10 | Hematologic malignancies/ 60, controls/ 18, cancer cell lines/ 2 | Retro | Frozen cells, bone marrow, peripheral blood | KT, FISH, CMA | 100 | Novel Tier 1 and 2 variants not previously reported by standard methods in 17 out of 60 (28%) cases | (10) |
| 11 | AML/ 159 | Prosp | Fresh blood or bone marrow | KT, FISH, targeted next-generation sequencing | 87 | Identified additional cytogenomic aberrations and/or provided information on fusion genes in 77 (48%) patients, including eight patients with normal KT and four with failed KT | (11) |
| 12 | MM/ 25 | Prosp | Bone marrow | FISH | 98.2 | ND | (12) |
| 13 | Prenatal samples/ 84 | Retro | Cultured amniocytes | KT, FISH, CMA | 100 | 64 additional clinically reportable SVs in 43 samples | (13) |
| 14 | Prenatal samples/ 37 | Prosp | Amniotic fluid, chorionic villi | KT, CMA | 100 | A median of 30 small (<100 kb) structural variations per case | (14) |
| 15 | Prenatal samples/ 34 | Retro | Amniotic fluid | KT, FISH, CMA | 97.8 | Determined the relative orientation and position of repetitive segments for seven cases with duplications or triplications | (15) |

**Supplementary Table 3 continued**

| No. | Study population/ Sample size (n) | Study design^a^ | Sample type | Conventional method^b^ | Concordance (%) | Additional findings | Ref. |
| --- | --- | --- | --- | --- | --- | --- | --- |
| 16 | Prenatal samples/ 204 | Prosp | Amniotic fluid | KT, CMA | KT (75.68), CMA (95.56) | Complex structural rearrangements and detailing the location and orientation of duplication CNVs | (16) |
| 17 | Prenatal samples/ 200 (123 unique cases) | Retro | Cryopreserved amniocytes or chorionic villus | KT, FISH, CMA | 99.6 | ND | (17) |
| 18 | Preconception samples/ 74 | Retro | Whole blood or amniotic fluid cells | KT, CMA | KT (94.7), CMA (100) | Improved the diagnostic rate by 5.4% and assisted in the diagnoses of six families (16.2%) | (18) |
| 19 | DD/ 49, RD/ 15, familial chromosomal aberrations/ 12, abnormal prenatal screening / 9 | Retro | Blood or cultured cells | KT, FISH, CMA | 100 | Complex rearrangements, including translocations t(3;4)(q13.11;q12), t(3;4)(q13.11;p11), and t(4;6)(q12;p22.3), inversion inv(13)(q31.2;q33.3) and chromoanagenesis | (19) |
| 20 | Unclear/ 14 | Retro | Peripheral blood, cell cultures | KT, FISH, CMA, MLPA | 85.7 | ND | (20) |
| 21 | Suspected genetic cases/ 359, controls/ 50 | Retro | Blood or cryopreserved cells | KT, FISH, CMA, Southern blot, PCR | 99.5 | 41 samples had additional clinically relevant findings or pertinent information | (21) |
| 22 | Genetic cases/ 55 | Retro | White blood cells or cultured cells | FISH, CMA | 100 | Refinement of the genomic structure in 12 cases | (22) |

**Supplementary Table 3 continued**

| No. | Study population/ Sample size (n) | Study design^a^ | Sample type | Conventional method^b^ | Concordance (%) | Additional findings | Ref. |
| --- | --- | --- | --- | --- | --- | --- | --- |
| 23 | Suspected genetic cases/ 76 | Retro | Frozen/fresh whole blood | KT, CMA, Southern blot | 98 | Provided diagnosis for three previously unsolved cases | (23) |
| 24 | Soft-tissue or bone tumors /38 | Retro | Snap-frozen biopsy specimens | KT, FISH | 91 | Detected a median of 206.5 SVs (500 bp to 5 Mb) per sample, ranging from 114 to 973 | (24) |
| 25 | Parkinson's disease/ 5 | Retro | Parkinson's disease-related iPSC and fibroblast line | ND | 100 | Two balanced inter-chromosomal translocations were detected in one line (iPS-L-3034) | (25) |
| 26 | RD/ 10 | Retro | Peripheral blood | KT, FISH, CNV-seq | 90 | ND | (26) |
| 27 | Neuromuscular disease/ 85 | Retro | Peripheral blood | PCR, RP-PCR, and Southern blot | 98.8 | Detected large repeat expansions with greater accuracy and indicated somatic repeat instability in 36 samples | (27) |

^a^ Retro, retrospective**;** Prosp, prospective;

^b^ CMA, chromosomal microarray analysis; CNV, copy number variation; FISH, fluorescence in situ hybridization; KT, karyotype; MLPA, multiplex ligation-dependent probe amplification; WES, whole exome sequencing; WGS, whole genome sequencing;

AML, acute myelocytic leukemia; ALL, acute lymphoblastic leukemia; BCP, B-cell precursor; CLL, chronic lymphocytic leukemia; DD, development delay; MDS, myelodysplastic syndromes; MM, multiple myeloma; RD, reproductive disorders;

ND**,** not described

**Reference**

1. Neveling K, Mantere T, Vermeulen S, Oorsprong M, van Beek R, Kater-Baats E, et al., Next-generation cytogenetics: Comprehensive assessment of 52 hematological malignancy genomes by optical genome mapping*.* Am J Hum Genet (2021) 108(8): 1423-35. doi: 10.1016/j.ajhg.2021.06.001.

2. Lestringant V, Duployez N, Penther D, Luquet I, Derrieux C, Lutun A, et al., Optical genome mapping, a promising alternative to gold standard cytogenetic approaches in a series of acute lymphoblastic leukemias*.* Genes Chromosomes Cancer (2021) 60(10): 657-67. doi: 10.1002/gcc.22971.

3. Rack K, De Bie J, Ameye G, Gielen O, Demeyer S, Cools J, et al., Optimizing the diagnostic workflow for acute lymphoblastic leukemia by optical genome mapping*.* Am J Hematol (2022) 97(5): 548-61. doi: 10.1002/ajh.26487.

4. Gerding WM, Tembrink M, Nilius-Eliliwi V, Mika T, Dimopoulos F, Ladigan-Badura S, et al., Optical genome mapping reveals additional prognostic information compared to conventional cytogenetics in AML/MDS patients*.* International Journal of Cancer (2022) 150(12): 1998-2011. doi: 10.1002/ijc.33942.

5. Balducci E, Kaltenbach S, Villarese P, Duroyon E, Zalmai L, Friedrich C, et al., Optical genome mapping refines cytogenetic diagnostics, prognostic stratification and provides new molecular insights in adult MDS/AML patients*.* Blood Cancer J (2022) 12(9): 126. doi: 10.1038/s41408-022-00718-1.

6. Sahajpal NS, Mondal AK, Tvrdik T, Hauenstein J, Shi H, Deeb KK, et al., Clinical Validation and Diagnostic Utility of Optical Genome Mapping for Enhanced Cytogenomic Analysis of Hematological Neoplasms*.* The Journal of Molecular Diagnostics : JMD (2022) 24(12): 1279-91. doi: 10.1016/j.jmoldx.2022.09.009.

7. Levy B, Baughn LB, Akkari Y, Chartrand S, LaBarge B, Claxton D, et al., Optical genome mapping in acute myeloid leukemia: a multicenter evaluation*.* Blood Advances (2023) 7(7): 1297-307. doi: 10.1182/bloodadvances.2022007583.

8. Brandes D, Yasin L, Nebral K, Ebler J, Schinnerl D, Picard D, et al., Optical Genome Mapping Identifies Novel Recurrent Structural Alterations in Childhood ETV6::RUNX1+ and High Hyperdiploid Acute Lymphoblastic Leukemia*.* Hemasphere (2023) 7(8): e925. doi: 10.1097/hs9.0000000000000925.

9. Shim Y, Koo Y-K, Shin S, Lee S-T, Lee K-A, and Choi JR, Comparison of Optical Genome Mapping With Conventional Diagnostic Methods for Structural Variant Detection in Hematologic Malignancies*.* Annals of Laboratory Medicine (2024) 44(4): 324-34. doi: 10.3343/alm.2023.0339.

10. Pang AWC, Kosco K, Sahajpal NS, Sridhar A, Hauenstein J, Clifford B, et al., Analytic Validation of Optical Genome Mapping in Hematological Malignancies*.* Biomedicines (2023) 11(12): 3263. doi: 10.3390/biomedicines11123263.

11. Loghavi S, Wei Q, Ravandi F, Quesada AE, Routbort MJ, Hu S, et al., Optical genome mapping improves the accuracy of classification, risk stratification, and personalized treatment strategies for patients with acute myeloid leukemia*.* American Journal of Hematology (2024) 99(10): 1959-68. doi: 10.1002/ajh.27435.

12. Yoon J, Kwon JA, and Yoon S-Y, Clinical Validation of Optical Genome Mapping in Multiple Myeloma Without Plasma Cell Enrichment*.* American Journal of Hematology (2025). doi: 10.1002/ajh.27589.

13. Sahajpal NS, Mondal AK, Fee T, Hilton B, Layman L, Hastie AR, et al., Clinical Validation and Diagnostic Utility of Optical Genome Mapping in Prenatal Diagnostic Testing*.* J Mol Diagn (2023) 25(4): 234-46. doi: 10.1016/j.jmoldx.2023.01.006.

14. Goumy C, Guy Ouedraogo Z, Soler G, Eymard-Pierre E, Laurichesse H, Delabaere A, et al., Optical genome mapping for prenatal diagnosis: A prospective study*.* Clinica Chimica Acta; International Journal of Clinical Chemistry (2023) 551: 117594. doi: 10.1016/j.cca.2023.117594.

15. Zhang Q, Wang Y, Xu Y, Zhou R, Huang M, Qiao F, et al., Optical genome mapping for detection of chromosomal aberrations in prenatal diagnosis*.* Acta Obstet Gynecol Scand (2023) 102(8): 1053-62. doi: 10.1111/aogs.14613.

16. Xie M, Zheng Z-J, Zhou Y, Zhang Y-X, Li Q, Tian L-Y, et al., Prospective Investigation of Optical Genome Mapping for Prenatal Genetic Diagnosis*.* Clinical Chemistry (2024) 70(6): 820-29. doi: 10.1093/clinchem/hvae031.

17. Levy B, Liu J, Iqbal MA, DuPont B, Sahajpal N, Ho M, et al., Multisite Evaluation and Validation of Optical Genome Mapping for Prenatal Genetic Testing*.* The Journal of Molecular Diagnostics : JMD (2024) 26(10): 906-16. doi: 10.1016/j.jmoldx.2024.06.006.

18. Yin K, Li M, Zhang H, Chang J, Qi Q, Zhou X, et al., Optical genome mapping to decipher the chromosomal aberrations in families seeking for preconception genetic counseling*.* Scientific Reports (2025) 15(1): 2614. doi: 10.1038/s41598-025-86828-9.

19. Mantere T, Neveling K, Pebrel-Richard C, Benoist M, van der Zande G, Kater-Baats E, et al., Optical genome mapping enables constitutional chromosomal aberration detection*.* Am J Hum Genet (2021) 108(8): 1409-22. doi: 10.1016/j.ajhg.2021.05.012.

20. Dremsek P, Schwarz T, Weil B, Malashka A, Laccone F, and Neesen J, Optical Genome Mapping in Routine Human Genetic Diagnostics-Its Advantages and Limitations*.* Genes (Basel) (2021) 12(12): 1958. doi: 10.3390/genes12121958.

21. Iqbal MA, Broeckel U, Levy B, Skinner S, Sahajpal NS, Rodriguez V, et al., Multisite Assessment of Optical Genome Mapping for Analysis of Structural Variants in Constitutional Postnatal Cases*.* J Mol Diagn (2023) 25(3): 175-88. doi: 10.1016/j.jmoldx.2022.12.005.

22. Barseghyan H, Pang AWC, Clifford B, Serrano MA, Chaubey A, and Hastie AR, Comparative Benchmarking of Optical Genome Mapping and Chromosomal Microarray Reveals High Technological Concordance in CNV Identification and Additional Structural Variant Refinement*.* Genes (2023) 14(10): 1868. doi: 10.3390/genes14101868.

23. Barseghyan H, Eisenreich D, Lindt E, Wendlandt M, Scharf F, Benet-Pages A, et al., Optical Genome Mapping as a Potential Routine Clinical Diagnostic Method*.* Genes (2024) 15(3): 342. doi: 10.3390/genes15030342.

24. Baelen J, Dewaele B, Debiec-Rychter M, Sciot R, Schöffski P, Hompes D, et al., Optical Genome Mapping for Comprehensive Cytogenetic Analysis of Soft-Tissue and Bone Tumors for Diagnostic Purposes*.* The Journal of Molecular Diagnostics : JMD (2024) 26(5): 374-86. doi: 10.1016/j.jmoldx.2024.02.003.

25. Trinh J, Schaake S, Gabbert C, Lüth T, Cowley SA, Fienemann A, et al., Optical genome mapping of structural variants in Parkinson's disease-related induced pluripotent stem cells*.* BMC Genomics (2024) 25(1): 980. doi: 10.1186/s12864-024-10902-1.

26. Dai P, Zhu X, Pei Y, Chen P, Li J, Gao Z, et al., Evaluation of optical genome mapping for detecting chromosomal translocation in clinical cytogenetics*.* Mol Genet Genomic Med (2022) 10(6): e1936. doi: 10.1002/mgg3.1936.

27. van der Sanden B, Neveling K, Shukor S, Gallagher MD, Lee J, Burke SL, et al., Optical genome mapping enables accurate testing of large repeat expansions*.* Genome Research (2025) 35(4): 810-23. doi: 10.1101/gr.279491.124.
